# Immune Evasion, Immunopathology and the Regulation of the Immune System

**DOI:** 10.3390/pathogens2010071

**Published:** 2013-02-13

**Authors:** Gabriele Sorci, Stéphane Cornet, Bruno Faivre

**Affiliations:** 1 Biogéosciences, UMR CNRS 6282, Université de Bourgogne, Dijon, France; E-Mail: bruno.faivre@u-bourgogne.fr (B.F.); 2 Maladies Infectieuses et Vecteurs : Ecologie, Génétique, Evolution et Contrôle (MIVEGEC), UMR CNRS 5290-IRD 224-UM1-UM2, Montpellier, France; E-Mail: stephan.cornet@gmail.com; 3 Centre d’Ecologie Fonctionnelle et Evolutive (CEFE), UMR CNRS 5175, Montpellier, France

**Keywords:** autoimmunity, immunosuppression, immune evasion, T_reg _cells, immune regulation, hygiene hypothesis, molecular mimicry

## Abstract

Costs and benefits of the immune response have attracted considerable attention in the last years among evolutionary biologists. Given the cost of parasitism, natural selection should favor individuals with the most effective immune defenses. Nevertheless, there exists huge variation in the expression of immune effectors among individuals. To explain this apparent paradox, it has been suggested that an over-reactive immune system might be too costly, both in terms of metabolic resources and risks of immune-mediated diseases, setting a limit to the investment into immune defenses. Here, we argue that this view neglects one important aspect of the interaction: the role played by evolving pathogens. We suggest that taking into account the co-evolutionary interactions between the host immune system and the parasitic strategies to overcome the immune response might provide a better picture of the selective pressures that shape the evolution of immune functioning. Integrating parasitic strategies of host exploitation can also contribute to understand the seemingly contradictory results that infection can enhance, but also protect from, autoimmune diseases. In the last decades, the incidence of autoimmune disorders has dramatically increased in wealthy countries of the northern hemisphere with a concomitant decrease of most parasitic infections. Experimental work on model organisms has shown that this pattern may be due to the protective role of certain parasites (*i.e.*, helminths) that rely on the immunosuppression of hosts for their persistence. Interestingly, although parasite-induced immunosuppression can protect against autoimmunity, it can obviously favor the spread of other infections. Therefore, we need to think about the evolution of the immune system using a multidimensional trade-off involving immunoprotection, immunopathology and the parasitic strategies to escape the immune response.

## 1. Introduction

In the last decade, evolutionary biologists interested in the ecology and evolution of host-parasite interactions and the evolution of parasite virulence have become more and more aware of the importance of immune defenses for the evolution of a number of host and parasite traits [1,2,3,4,5]. This has lead to the emergence of a novel discipline named “ecological immunology” [6]. A central tenet of this discipline is that maximal investment into immune defenses might not be synonymous of optimal strategy, because maximal defenses do not necessarily translate into maximal fitness [7]. The reason for this is that investment into immune defenses can be afforded at the expense of investment into other fitness-related functions, which generates trade-offs between traits [8,9]. This sets a clear analogy between immune defenses and any other life history trait. As maximal investment into reproduction might not be an optimal strategy for an iteroparous species, because of the survival costs of breeding effort [10], maximal investment into immune defenses might come at cost of investment into other life history traits, such as growth rate, reproductive output or other physiological functions related to self-maintenance [11,12]. The general paradigm that has been adopted by ecological immunology studies is that these trade-offs are generated because immune defenses are costly in terms of energy and/or limited resources [13]. This paradigm has attracted considerable attention, and several studies have attempted to assess the energetic cost of immune activation [14,15,16,17,18,19,20]. Although there is a general consensus on the idea that the immune response requires metabolic resources, the question whether the magnitude of the cost is sufficient to force the emergence of trade-offs is still debated [15,19,20].

However, immunity does not entail only energetic costs. The immune system is often depicted as a two-edged sword, where one edge protects against infectious diseases and the other edge exposes to the risk of misdirected immune reaction. This phenomenon is generally called immunopathology and illustrates the process of immune system attacking the structures of the host. Immune-mediated diseases can be classified according to two main modes of action: antigen-specific and antigen-nonspecific [21]. An erroneous recognition of the antigen that triggers the immune response can induce substantial costs for the host, depending on the molecule/organ that is the target of the misoriented immune reaction. A classic example of immune-mediated diseases is type1 diabetes mellitus (T1DM), where T-cells and antibodies progressively target and destroy β cells in the islets of Langerhans in the pancreas and the hormone these cells secrete (*i.e.*, insulin) [22]. Antigen-nonspecific immunopathology arises as a collateral undesirable effect of an over-reacting immune response against an invading parasite, for instance, during chronic inflammation. Immunopathology is actually thought to be one of the principal determinants of the most serious symptoms induced by some human infectious diseases, such as tuberculosis, malaria or the highly pathogenic influenza virus [23,24]. Similarly, studies on animal models, both vertebrates and invertebrates, have provided a wealthy of evidence on the dual effect of the immune system and the cost of immunopathology (*i.e.*, [25,26]).

Since an over-reacting immune response—or a response failing to make a distinction between self and non-self―potentially generates substantial fitness costs [27,28], one should expect natural selection to have favored the evolution of regulation mechanisms that prevent the system to get out of control. Indeed, the immune system is characterized by a series of checkpoints and filters that block and prevent the spread of autoreactive cells and shape immunological tolerance (*i.e.*, the nonreactivity of the immune system to a given antigen). This regulation includes central (the negative selection of self-reactive lymphocytes in the thymus) and peripheral mechanisms (anergy, activation of regulatory T-cells) [22]. In spite of this regulatory network, autoimmune disorders are common diseases. From an evolutionary perspective this might seem puzzling, since natural selection should progressively favor individuals with the most effectively regulated immune system. In this article, we suggest that the maintenance of immunopathology by natural selection might be due to the combined effect of two factors: (i) the trade-off between immunoprotection and immunopathology and (ii) the strategies adopted by parasites to escape and manipulate the host immune response. 

We will first briefly review the principal effectors that regulate the immune response, and we will illustrate the dilemma faced by the immune system between protection and over-reaction; then, we will see how parasitic strategies of immune evasion can alter up- and down-regulatory mechanisms and, as such, exacerbate or protect hosts from immunopathology. Finally, we will stress that all the empirical results that we discuss here come from studies on humans and model organisms. The relevance of what is going on in modern human societies and congenic strains of mice with respect to natural conditions is open to discussion. 

## 2. The Regulation of the Immune System

The immune system is a sophisticated and complex weapon that has evolved to destroy invading pathogens [29]. Benefits of having a well-performing immune system are clearly shown by the pathologies associated with congenital and/or acquired immunodeficiencies. The protective function of the immune system resides in the capacity of immune cells to discriminate between self and non-self antigens. Major histocompatibility complex (MHC) molecules expressed on the surface of all nucleated cells (class I), and “professional” antigen presenting cells (class II) are an essential tool for the recognition of non-self antigens. These proteins bind to antigenic peptides and present them to T-cells. 

CD4^+^ T-cells play a pivotal role in the polarization of the immune response. Following a microbial stimulus, naive CD4^+^ T-cells differentiate into Th1, Th2 and T_reg_ helper cells. These cells are characterized by the cytokines they produce. A Th1 response is characterized by the production of pro-inflammatory cytokines, such as gamma interferon (IFN-γ) and tumor necrosis factor alpha (TNF-α), which activate cell-mediated reactions. The Th1 response is usually elicited by many intracellular pathogens. The Th2 response is, on the contrary, characterized by the production of a different set of cytokines (IL-4, IL-5, IL-10, IL-13), which are responsible for immunoglobulin class switching and the inhibition of inflammation. Interestingly, cytokines of the Th1 phenotype tend to inhibit Th2 effector functions, whereas cytokines of the Th2 phenotype inhibit Th1 functions. T_reg _cells are characterized by the CD25^+^ receptor and account for 5-10% of peripheral CD4^+^ T-cells in mouse and humans. T_reg _cells contribute to ensure self-tolerance by suppressing autoreactive lymphocytes, mostly through contact or via the secretion of anti-inflammatory cytokines, such as IL-10 and transforming growth factor beta (TGF-β) [30,31,32].

Any dysfunction of the immune system exposes the host to several potential costs, such as an increased risk to contract an infection or suffer from an autoimmune disorder. This emphasizes the need of control mechanisms that regulate the functioning of immune effectors [33]. Control mechanisms that prevent the spread of autoreactive T-cells operate in the thymus by the deletion of immature T-cells that recognize self-antigens and in peripheric organs by anergy, deletion by apoptosis and the action of the above mentioned T_reg _cells [34,35,36,37]. Whereas deletion of immature T-cells in the thymus limits the risk of antigen-specific immunopathology, mechanisms that operate in peripheric organs contribute to the control of both antigen-specific and antigen-nonspecific immunopathology. In the rest of this review, we will focus on the control mechanisms operating in peripheric organs. 

Much attention has been recently devoted to T_reg _cells as an essential component of the regulatory network that avoids the over expression of the immune response. The paramount role of T_reg _lymphocytes has been established with a series of elegant experiments. Mutation in the gene encoding the transcription factor Foxp3, which is required for T_reg _lymphocyte development, induces lethal autoimmunity [38]. Similarly, *in vivo* ablation of T_reg _cells produces catastrophic autoimmunity in both juvenile and adult mice [30].

Given that autoreactive T- and B-cells, as well as cells of the inflammatory response are part of the normal immune cell repertoire in healthy individuals, the key point to understand autoimmunity is to identify the factors that trigger these quiescent autoreactive cells to actively target host tissues and organs, in spite of the regulatory mechanisms. The occurrence of autoimmune diseases is not a rare event, in particular in western societies, where the incidence of some autoimmune disorders has dramatically increased in the last decades [39,40,41,42]. Then, why natural selection has not succeeded to eliminate immune-related diseases or keep them at a low rate?

## 3. Immune Evasion, Immune Regulation and Immunopathology

**Figure 1:**
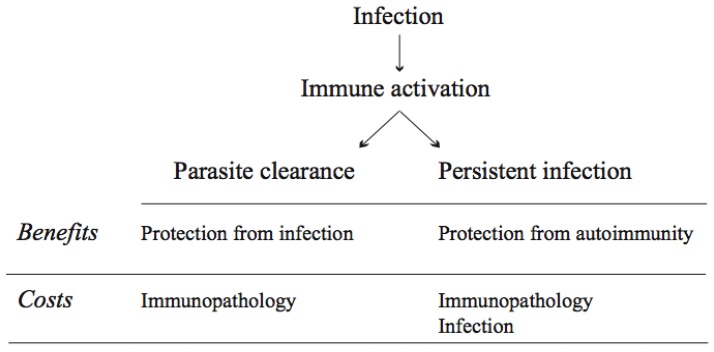
A schematic view of the costs and benefits of infection-induced immune activation. Although immune mediated parasite clearance has evident benefits in terms of parasite resistance, it can also induce costs in terms of immunopathology. Similarly, although failure to clear the parasite and the persistent infection that ensues are usually considered to be costly for the host, it can also generate benefits in terms of protection from autoimmunity.

If pathogens were static entities, the regulatory mechanisms that avoid the overexpression of the immune response might be enough to ensure the optimal functioning of the system. However, pathogens are evolving entities and, as such, they do respond to the selection pressures exerted by the immune system. One might even think of a successful parasite as a parasite that has escaped the immune response of the host, at least until transmission to a novel host. Immune evasion is a common strategy of parasitic organisms, including microparasites (viruses, bacteria and protozoa), as well as metazoan helminths [43,44,45,46,47,48]. Immune evasion can take several forms, as hiding from and suppressing the immune response [49]. Therefore, parasite interference with the normal immune response poses a novel problem to the immune system, since parasite manipulation of the immune response is likely to alter the balance that exists between immunoprotection and immunopathology (Figure 1).

### 3.1. Immune Suppression

Although immune evasion has been selected to favor parasite establishment within the host, it is likely that some particular strategies adopted to escape the immune response might paradoxically be beneficial also for the hosts [42,50]. In particular, parasitic strategies that dampen immune reactivity, while being clearly favorable for the parasite, could also protect the host from immunopathology [21,51,52,53,54,55]. Is there any evidence that this might be the case? 

Helminths are multicellular worm parasites that usually occur with high prevalence in natural populations. In many instances, the infection persists in face of the host immune response, suggesting that helminths have evolved effective strategies of immune evasion and suppression [42,56]. Direct experimental evidence for the role played by helminths in the protection from immunopathology comes from several studies involving different helminth parasites of mice [57,58]. For instance, experimental infection with the trematode *Schistosoma mansoni* prevents the development of T1DM in non-obese diabetic (NOD) mice [59], as well as allergic encephalomyelitis [60] and Grave’s thyroiditis [61]. Intestinal nematodes, such as the murine parasite *Heligsomoides polygyrus*,have also been shown to have immunomodulator properties. Mice with chronic *H. polygyrus* infections are less likely to develop airway inflammation [62] and allergy [63].

Given that helminths induce a Th2 type response, the protective role of such infections was first explained with the Th1/Th2 paradigm [64]. Th2 cytokines inhibit the Th1 effectors thought to be the principal cause of inflammatory and immune-mediated diseases. Recently, however, evidence has accumulated showing the involvement of T_reg_ cells [56]. In addition to helminths, other pathogens can also protect from autoimmune diseases by activating T_reg _cells [65]. For instance, *Leishmania major* [66,67], herpes simplex virus type 1 [68], murine leukemia virus [69] and certain strains of *Plasmodium yoelii* [70] have all been shown to escape the host immune system by activating T_reg _cells. 

Parasitic strategies to escape the immune response also involve dampening of inflammation, a major determinant of immunopathology. Some pathogens can secrete molecules that interfere with the signaling pathways triggering the inflammatory response, such as the Nuclear Factor kappaB (NF-*k*B) (a transcription factor that plays a key role in regulating the immune response to infection) or cytokine IL-10. Enterobacteria from the genus *Yersinia* produce a protease that downregulates the NF-*k*B signaling pathway [71]. A few intracellular pathogens (e.g., HIV, cytomegaloviruses, mycobacteria) can dampen the host immune response by inducing the production of the anti-inflammatory cytokine IL-10 by the host or by expressing a IL-10 homolog [72]. Interestingly, these pathogens likely benefit from the IL-10 driven immunosuppression, as well as by other mechanisms unrelated to the anti-inflammatory action of IL-10. For instance, IL-10 has been shown to increase the expression of the CCR5 receptor, a major co-receptor for HIV-1 [73]. Similarly, cytomegalovirus-induced production of IL-10 interferes with the antigen-presentation pathway by downregulating MHC class II expression [74]. 

Inducible nitric oxide synthase (iNOS) also plays a primordial role in inflammation and pathogen killing by producing RNS, such as nitric oxide (NO). Some pathogens have evolved a strategy to prevent the production of NO in macrophages by repressing or inhibiting iNOS gene expression [47]. For instance, *Citrobacter rodentium*, an intestinal parasite of mice, inhibits iNOS activity in the intestinal region, where the infection is localized, although the activity of the enzyme can be upregulated in other organs [75]. 

To conclude, whereas, on one hand, immune suppression directly benefits the parasite to persist within the host and establish a chronic infection, the parasite-induced downregulation of the immune response can also be beneficial for the host in terms of reduced immunopathology risk, as illustrated by the clinical trials using nematode worms to cure Crohn’s disease and ulcerative colitis [76,77].

### 3.2. Molecular Mimicry

As already said, immunopathology is often seen as an unavoidable cost of immune protection, something that harms the host, with no obvious benefit for the parasite. This might not always be true, since strategies of immune evasion that enhance parasite survival within the host can directly be responsible for the onset of immune disorders. Parasitic strategies to escape immune defenses can, therefore, protect from or exacerbate immunopathology [78] (Table 1). Molecular mimicry is one such mechanism that might favor the pathogen [79], while increasing the risk of autoimmune disorders. Molecular mimicry refers to the property of a given pathogen to share antigenic determinants with the host [80,81,82,83]. Pathogens that mimic host antigens can evade immunity, because self-tolerance mechanisms eliminate or anergize autoreactive T-cells. However, depending on the degree of structural resemblance between parasite and host epitopes and/or the repeated activation of autoreactive T-cells during infection, mimicry can stimulate the destruction of host tissues. Herpes simplex virus 1-infected mice develop herpes stroma keratitis, because autoreactive T-cells attack corneal tissue. The reason for this autoimmune reaction is a cross-reactive epitope between the virus (the UL6 protein) and a host corneal protein. Strong support to the view that stroma keratitis is due to molecular mimicry comes from experiments where viruses lacking the UL6 protein or expressing an altered mimic epitope are inoculated [84]. In this case, mice do not develop the autoimmune disorder. Another example of immunopathology induced by molecular mimicry is provided by Guillain-Barré syndrome, an autoimmune disease affecting the nervous system that is usually induced by infection with the bacterium *Campylobacter jejuni* [85,86]. Indeed, it has been shown that *C. jejuni* expresses a lipopolysaccharide that imitates gangliosides found in peripheral nerves [87].

Although molecular mimicry is an appealing hypothesis to account for the persistence of immunopathology by natural selection, we should emphasize that, since the concept was put forward by Damian [79], there is very few direct evidence showing that molecular mimicry has evolved as a strategy to allow the persistence of the parasite within the host. Theoretical and empirical work exploring the fitness consequences of molecular mimicry is definitely needed [88]. Herpes simplex virus 1 offers the possibility to explore the fitness consequences of molecular mimicry, since one should be able to compare the fitness of the strains expressing the UL6 protein *vs.* those that do not or that express a slightly altered epitope. 

**Table 1:** A non-exhaustive list of parasites (viruses, bacteria, protozoa and helminths) that have been reported to protect and/or induce immunopathology. Only experimental evidence is reported here. IBD = Inflammatory bowel disease; T1DM = Type 1 diabetes mellitus.

Parasite	Protection	Exacerbation	Ref.
**Viruses**			
Influenza A virus		Allergy	[89]
Influenza A virus		Airway responsiveness	[90]
Respiratory Syncytial Virus		Airway hyper-reactivity	[91]
Respiratory Syncytial Virus	Asthma		[92]
Respiratory Syncytial Virus		Allergy	[93]
B3 Coxsackie virus		Myocarditis	[94]
B3 Coxsackie virus		T1DM	[94]
Rubella virus		T1DM	[94]
Rhinovirus		Allergy	[95]
Mouse hepatitis virus	T1DM		[96]
Mouse adenovirus type 1		Encephalomyelitis	[25]
Herpes simplex virus		Keratitis	[84]
**Bacteria**			
*Mycobacterium bovis*	Encephalomyelitis		[97]
*Mycobacterium bovis*	Multiple sclerosis		[98]
*Mycobacterium bovis*	T1DM		[99]
*Mycobacterium tuberculosis*		Adjuvant arthritis	[100]
*Mycobacterium avium*	T1DM		[101]
*Mycobacterium avium*	Systemic lupus erythematosus		[102]
*Mycobacterium vaccae*	Allergy		[103]
*Salmonella typhimurium*	T1DM		[54]
*Chlamydia trachomatis*	Allergy		[104]
*Borrellia burgdorferi*		Lyme arthritis	[105]
*Bordetella pertussis*		Allergy	[106]
*Bordetella pertussis*	Encephalomyelitis		[107]
*Mycoplasma pneumoniae*		Airway hyper-reactivity	[108]
*Staphylococcus aureus*		Allergy	[95]
*Streptococcus sanguinis*	Collagen-induced arthritis		[109]
*Streptococcus A*		Rheumatic heart disease	[110]
*Streptococcus pyogenes*		Rheumatic fever	[111]
*Helicobacter pylori*		Autoimmune gastritis	[81]
*Campylobacter jejuni*		Guillain-Barré syndrome	[85]
**Protozoa**			
*Trypanosoma cruzii*		Chaga’s disease	[94]
*Trypanosoma brucei*	Collagen-induced arthritis		[112]
**Helminths**			
*Hymenolepis diminuta*	Colitis		[113]
*Heligmosomoides polygyrus*	IBD		[114]
*Schistosoma mansoni*	T1DM		[115]
*Schistosoma mansoni*	T1DM		[59]
*Schistosoma mansoni*	Colitis		[116]
*Schistosoma mansoni*	Encephalomyelitis		[60]
*Schistosoma mansoni*	Hyperthyroidism		[61]
*Trichuris suis*	Chron’s disease		[76]
*Trichuris suis*	IBD		[117]
*Trichinella spiralis*	Colitis		[118]
*Heligmosomoides polygyrus*	Allergy		[63]
*Nippostrongylus brasiliensis*	Allergy		[119]
*Nippostrongylus brasiliensis*		Airway hyper-responsiveness	[120]
*Strongyloides stercoralis*	Allergy		[121]
*Brugia malayi*		Airway hyper-responsiveness	[122]

## 4. The “Educational” Role of Antigenic Exposure

In addition to experimental work on animal models, the potential role played by the coevolutionary interactions between hosts and parasites in shaping patterns of immune regulation can also be apprised by the means of epidemiological studies. Epidemiological studies have clearly established the existence of a North-South cline in the prevalence of many autoimmune disorders and that in western, wealthy societies, the incidence of autoimmune diseases has increased tremendously in the last decades [123]. The high rate of increase [40], as well as the observation that migrating people acquire the profile risk of resident populations [39], concurs to exclude a major genetic determinism for these changes for most autoimmune disorders. Instead, it has been argued that the spatial and temporal variation in the occurrence of autoimmune disease could be linked to the variation in infection risk [124]. Early observations [125] showed that the occurrence of hay fever was negatively correlated with family size, possibly because late born children are exposed to a number of infectious diseases, carried by the first born child, that protect them from allergy. The idea underlying this so-called “hygiene hypothesis” is that the immune system has evolved and has been selected to cope with a diversity of surrounding parasitic organisms, each of which plays a role in the “education” (up-/down-regulation) of the immune response [126]. Human modern lifestyle has profoundly modified the interactions between the immune system and the pathogens currently faced. The eradication of helminths, the societal pressures to have clean habitats, the widespread use of antibiotics have, of course, greatly contributed to the improvement of human health, but perhaps we are now starting to pay the cost of these changes in terms of increased risk of autoimmune disease [41,53,57,123]. In line with this view, trial assays have been performed with the parasitic nematodes, *Trichuris suis* and *Necator americanus*, as “therapeutic” agents against inflammatory bowel disease and asthma [76,127].

Investigations of the “hygiene hypothesis” have also taken advantage of the particular environmental conditions encountered in farms to assess the role played by microbial exposure on both the innate and adaptive immune system and the subsequent risk to develop autoimmune disorders. Comparative epidemiological studies conducted on groups of children living either in farms or in cities have provided a number of associations between exposure to lipopolysaccharide (LPS) of the cell wall of Gram-negative bacteria (farm children are exposed to higher dose of LPS in mattress dust) and allergen sensitization, hay fever and asthma [128,129,130]. These results suggest that living in a farm protects from some autoimmune disorders, although it must be stressed that the protective effect of LPS on future risk of allergy also depends on a number of factors, such as the timing of exposure (early *vs*. late exposure), the intensity of antigenic exposure, the nature of co-existing antigenic exposures and polymorphism of genes responsible for the immune response against LPS.

Although there are several studies that have reported consistent epidemiological evidence for a positive association between a number of indices linked with infection risks [126,131], including socioeconomic factors [132,133], and the incidence of autoimmune disorders, we should keep in mind that such correlative work fails to establish a causal link between infection and protection from autoimmunity [134]. The role of confounding behavioral factors has been emphasized has a major challenge for these epidemiological surveys [135]. In spite of these criticisms, it should be noted that more direct, experimental evidence in support for the hygiene hypothesis comes from studies on animal models. The idea that living in too clean habitats might exacerbate the risk of autoimmunity receives support from the observation that animals raised in a germ-free environment develop autoimmune diseases faster and at higher rate than similar strains living in conventional environments [39].

## 5. Predicting the Evolutionary Consequences of Immune Evasion on Immune Regulation

As emphasized above, the classic view on the evolution of immune defenses has been that conflicting demands of energy consuming functions shape the allocation of resources to each of them as to maximize fitness [6,136]. However, acknowledging the role of parasitic strategies to escape the immune system costs can broaden the focus from the resource allocation paradigm to the immune regulation hypothesis. If an hyperactive immune system can harm the host, the important trait likely to be under strong selection pressure is not only the total amount of energy/resources allocated to immune organs and cells, but also the effectiveness of the regulatory mechanisms that prevent the system to get out of control [137,138]. Bergstrom and Antia [33] have already discussed the importance of taking into account the need to avoid autoimmunity and to prevent parasite sabotage if we want have a better understanding of the complexity of the immune system. 

Low total investment into immune organs with a lack of regulatory effectors can be more harmful than a huge allocation into immune defenses with a very effective immune regulation. Focusing on the benefits and costs of immune regulation, one can figure out the optimal level of immune regulation. Benefits are likely to increase with the level of immune regulation, because regulation prevents immunopathology, until a plateau is reached (once the risk of immunopathology is nil, no further benefit is expected from more regulation). On the contrary, the cost function should be “U-shaped”, with a weakly regulated immune system exposing the host to high risk of immunopathology and a highly regulated immune system being unable to clear the infection.

Parasites can, however, alter the shape of the cost/benefit functions. Parasites that protect from immunopathology reduce the cost of a weakly regulated immune system and should select for less regulated immune responses. Conversely, parasites that exacerbate autoimmunity should favor hosts with a highly regulated immune system. 

In a similar way, we might wonder how parasitic strategies that have evolved to face the host immune system—such as parasite induced immunosuppression—would affect host fitness [139]. While it is straightforward to think that parasite-induced immunosuppression is beneficial to the parasite, the effect on host fitness can be quite different, depending on the ratio between the cost of infection and the protection from immunopathology. When the cost of infection is low and/or the benefits in terms of protection from autoimmunity high (*i.e.*, low values of the ratio between cost of infection and protection from autoimmunity), we can end up with a flat fitness function for the host. This could be the case for many helminth infections, where the parasites persist for long time within the host with little effect on host fitness. On the contrary, when the cost of infection is high and/or the protection from immunopathology marginal (*i.e.*, high values of the ratio between cost of infection and protection from autoimmunity), the fitness function can be quite steep.

Of course, this is a crude verbal representation of the possible relationships between parasitic strategies, immune regulation and host and parasite fitness, and quantitative models are needed to have more clear-cut predictions (see, for instance, [140,141]). Nevertheless, we believe that this verbal model shows the importance to broaden our view on the evolution of the immune response (i) by taking into consideration parasitic strategies and risk of immunopathology in addition to the classical energetic costs of immune functioning and (ii) by thinking about the evolution of the immune system using a multidimensional trade-off involving immunoprotection, immunopathology and the parasitic strategies to escape the immune response.

Frank and Schmid-Hempel [142] provided an interesting hypothesis on the possible evolutionary consequences of immune evasion for parasite virulence. They drew a parallel with life history theory and showed that mechanisms that improve the survival of the pathogen within the host (by escaping the host immune response) have greater fitness sensitivity than mechanisms that incrementally alter transmission. This suggests that immune evasion mechanisms can result in increased pathogenic effects and affect virulence evolution. These ideas have further been developed by taking into account the time lag between the benefit of the immune evasion mechanism and the pathogenic effect (the longer the time lag, the strongest the selection for higher virulence) and the spread of the immune evasion mechanism throughout the host that should select against virulence (compared to a localized immune evasion mechanism) [48]. These theoretical models provide nice testable predictions that should elicit interest from experimentalists working on the evolution of parasite virulence.

In addition to theoretical work, we suggest that artificial selection experiments and experimental coevolution studies might provide very relevant insights into the outcome of evolutionary “contest” between host immune protection/regulation and parasite immune evasion. For instance, using mice and parasites known to protect from or induce immunopathology, one should be able to measure the evolutionary response in terms of T_reg_ cell numbers and production of anti-inflammatory cytokines (*i.e.*, IL10, TGF-β).

As we mentioned above, some countries of the northern hemisphere have experienced a significant reduction of antigenic stress, due to the improved hygienic conditions, but a concomitant increase in the incidence of autoimmune diseases. Although we have to acknowledge the potential effect of confounding socio-economic factors, we might wonder whether these changes cannot be seen as a large scale experiment on the evolution of immune regulation under variable antigenic contexts. 

## 6. Limits

Although ecological immunology has become a very fecund field of investigation, the fitness consequences of immune overreaction and how immune evasion interferes with optimal investment into the immune function have been less studied. There are, of course, several reasons for this, the most important being the difficulty to assess immunopathology (but, see [143,144,145]) and immune evasion in non-model, free-ranging species. Studying the evolutionary ecology of the immune response implies assessing the evolutionary forces and the environmental constraints that shape the investment into immune defenses, and this can only be done with outbred, free-ranging organisms that are exposed to a wide diversity of parasites and variable environmental conditions.

We need to emphasize that all the evidence for immunopathology and associated risk-enhancing and risk-reducing effects of infection comes from studies carried out on humans and animal models. The extension of these results to natural populations of free-ranging organisms might, therefore, not be straightforward. Autoimmune disorders are multifactor, complex diseases that are triggered by several genetic and environmental factors, in an additive and, often, in an interactive way [146]. The timing of onset of immunopathology during an individual lifespan is obviously an important determinant of the fitness consequences of autoimmunity. Autoimmune disorders can be expressed during the early phase of development and can rapidly result in the death of the individual. Genetic variants that are associated with an accrued risk to develop an autoimmune disease [147] should be strongly counter-selected and should participate very little to the polymorphism observed at the population level. T1DM has a juvenile onset and is under the control of many genes in humans and rodents and was certainly lethal, until the discovery of effective treatment with insulin by Banting and Best ([148] in [40]). Contrary to the expectation that natural selection should remove these deleterious genetic variants from the present-day populations, these genes persist [147]. The reasons for this are unknown, but one might speculate that either the genes predisposing to diabetes or genes in linkage disequilibrium have been retained, because they convey a selective advantage. Alternatively, in line with the rationale of the “hygiene hypothesis”, infections “neutralized” the deleterious expression of such genes, until recently, when the eradication of most endemic infectious diseases has allowed the full expression of alleles predisposing to autoimmunity. 

At the other extreme of the gradient, some autoimmune disorders have a delayed timing of onset, with several years elapsed between the triggering effect (for instance, infection) and the development of the disease. If such delay is also necessary for the onset of autoimmune disorders in natural settings, the relevance of autoimmunity costs for the evolution of optimal immune functioning would be dramatically reduced, simply because, depending on the specific life history strategy, the immunopathology cost might concern a relatively small fraction of the population. Similarly, autoimmunity that is triggered by a dysfunction of the effectors of inflammation and that is typical of age-related diseases in humans [149] might be of little relevance for natural populations, because of the decline in the intensity of natural selection with age [150]. This obviously reduces the spectrum of potential autoimmune diseases that might play a significant evolutionary role in shaping the pattern of optimal allocation into immune defenses. 

Free-ranging organisms are exposed to a wide variety of pathogens and parasites, which makes it difficult to unambiguously identify the causative agent of an autoimmune disorder. Moreover, pathogens that simultaneously co-infect a host can interfere with the immune response mounted against other antigens. This has been fairly well demonstrated in several experimental infections of animal models [151,152]. For instance, it has been shown that latent infection with herpesviruses confers resistance to mice infected with two bacterial pathogens (*Listeria monocytogenes* and *Yersinia pestis*) [153]. After a series of lytic replication in a number of cell types, herpesvirus enters a dormant state in B-cells, macrophages and dendritic cells. Barton *et al*. [153] reported that latent herpesvirus infection induces a persistent activation of macrophages (including of those that are not actually infected by the virus) with a subsequent elevated level of pro-inflammatory cytokines, such as IFN-γ and TNF-α. Although a permanently enhanced secretion of pro-inflammatory cytokines can induce a benefit for the host in terms of resistance to bacterial infection, it can also expose it to risk of autoimmune disorders and cancer. Whether this type of immune-mediated interspecific interactions is widespread or rare under natural conditions is unknown, as is unknown whether these effects are the side-product of the interaction between the parasite and the host immune system or whether they are of any adaptive value for the parasite. 

These examples document the difficulty to establish an unequivocal and general causal link between infectious diseases, strategies that pathogens adopt to escape the host immune system and autoimmune disorders. 

## 7. Conclusions

Ecological immunology is a relatively novel discipline that has its roots in a few seminal papers that have been published during the early 90s. In fifteen years, we have witnessed a growing interest from evolutionary biologists to the role played by the immune system for the ecology and evolution of host-parasite interactions. We believe that this early phase of the emergence of a new discipline is behind us, and we need now to go a step further by establishing a tighter link between the evolutionary approach and the knowledge of the immunological mechanisms underlying the control of the immune response in the face of rapidly evolving pathogens. 
